# 3,14-Dimethyl-2,6,13,17-tetra­aza­tricyclo­[16.4.0.0^7,12^]docosa­ne–(naphthalen-1-yl)methanol (1/2)

**DOI:** 10.1107/S160053681105272X

**Published:** 2011-12-14

**Authors:** Jong-Ha Choi, Md Abdus Subhan, Keon Sang Ryoo, Seik Weng Ng

**Affiliations:** a Department of Chemistry, Andong National University, Andong 760-749, Republic of Korea; b Department of Chemistry, University of Malaya, 50603 Kuala Lumpur, Malaysia; c Chemistry Department, Faculty of Science, King Abdulaziz University, PO Box 80203 Jeddah, Saudi Arabia

## Abstract

In the title co-crystal, C_20_H_40_N_4_·2C_11_H_10_O, the macrocycle is generated by a crystallographic inversion centre. The N atoms show a pyramidal coordination, and the cyclo­hexane ring that is fused to the 14-membered C_10_N_4_ ring exists in a chair conformation, whereas the methyl substituent occupies an axial site. The (naphthalen-1-yl)methanol mol­ecule forms an O—H⋯N hydrogen bond to a cyclam N atom. The mean-square-plane passing through the 14-membered ring is approximately coplanar with the naphthalene fused-ring [dihedral angle = 6.6 (1)°].

## Related literature

For the synthesis of the cyclam, see: Kang & Jeong (2003 ▶).
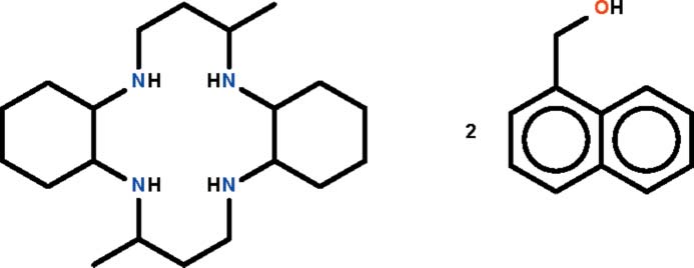


## Experimental

### 

#### Crystal data

C_20_H_40_N_4_·2C_11_H_10_O
*M* *_r_* = 652.94
Triclinic, 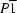

*a* = 8.9706 (4) Å
*b* = 9.4967 (6) Å
*c* = 10.5580 (5) Å
α = 92.500 (4)°
β = 97.961 (4)°
γ = 96.666 (4)°
*V* = 883.13 (8) Å^3^
*Z* = 1
Mo *K*α radiation
μ = 0.08 mm^−1^
*T* = 100 K
0.30 × 0.25 × 0.20 mm

#### Data collection

Agilent SuperNova Dual diffractometer with an Atlas detector
Absorption correction: multi-scan (*CrysAlis PRO*; Agilent, 2010 ▶) *T*_min_ = 0.978, *T*_max_ = 0.985
6926 measured reflections
3915 independent reflections
2958 reflections with *I* > 2σ(*I*)
*R*_int_ = 0.027

#### Refinement

*R*[*F* ^2^ > 2σ(*F* ^2^)] = 0.049
*wR*(*F* ^2^) = 0.126
*S* = 1.00
3915 reflections
229 parameters
3 restraints
H atoms treated by a mixture of independent and constrained refinement
Δρ_max_ = 0.29 e Å^−3^
Δρ_min_ = −0.28 e Å^−3^

### 

Data collection: *CrysAlis PRO* (Agilent, 2010 ▶); cell refinement: *CrysAlis PRO*; data reduction: *CrysAlis PRO*; program(s) used to solve structure: *SHELXS97* (Sheldrick, 2008 ▶); program(s) used to refine structure: *SHELXL97* (Sheldrick, 2008 ▶); molecular graphics: *X-SEED* (Barbour, 2001 ▶); software used to prepare material for publication: *publCIF* (Westrip, 2010 ▶).

## Supplementary Material

Crystal structure: contains datablock(s) global, I. DOI: 10.1107/S160053681105272X/hg5151sup1.cif

Structure factors: contains datablock(s) I. DOI: 10.1107/S160053681105272X/hg5151Isup2.hkl

Supplementary material file. DOI: 10.1107/S160053681105272X/hg5151Isup3.cml

Additional supplementary materials:  crystallographic information; 3D view; checkCIF report

## Figures and Tables

**Table 1:** Hydrogen-bond geometry (Å, °)

*D*—H⋯*A*	*D*—H	H⋯*A*	*D*⋯*A*	*D*—H⋯*A*
O1—H1o⋯N1	0.85 (1)	1.94 (1)	2.786 (2)	171 (2)

**Fig. 1.**
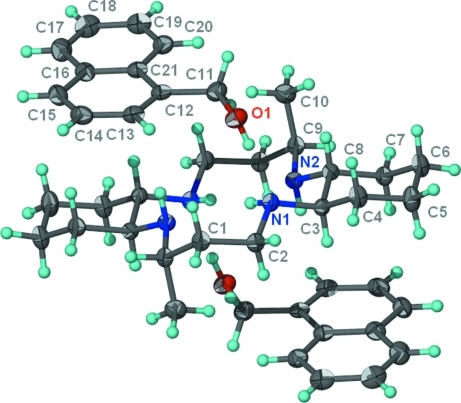
Thermal ellipsoid plot (Barbour, 2001) of C20H40N4.2C11H10O at the 70% probability level; hydrogen atoms are drawn as spheres of arbitrary radius.

## supplementary crystallographic information

### Comment

We had intended to react the cyclam having 1,2-diaminocyclohexanediamine sub-units, 5,16-dimethyl-2,6,13,17-tetraazatricyclo[14,4,0^1,18^,0^7,12^]docosane, with an alkyl chloride to form the corresponding ammonium salt; however, under the basic conditions, the 1-chloromethyl-naphthalene component was hydrolyzed to 1-hydroxymethyl-naphthalene, which co-crystallizes with the cyclam (Scheme I, Fig. 1). The cyclam lies on a center-of-inversion, and the nitrogen atoms of the 14-membered C_10_N_4_ ring show pyramidal coordination. However, these are not enaged in any hydrogen-bonding interactions. The cyclohexane rings that are fused to the 14-membered ring exist in chair conformations, and the methyl substituents in axial configurations.

The 1-hydroxymethyl-naphthalene molecule forms an O–H···N hydrogen bond to the cyclam (Table 1).

The mean-square-plane passing through the 14-membered ring is approximately co-planar with the naphthalene fused-ring (dihedral angle 6.6 (1) °).

### Experimental

The macrocycle 3,14-dimethyl-2,6,13,17-tetraazatricyclo[16.4.0.0^7,12^]docosane was synthesized by using a published procedure (Kang & Jeong, 2003). To a solution of this marcrocycle (0.61 g, 2.0 mmol) in methanol (10 ml) was added 1-chloromethylnaphthalene (0.80 g, 4.53 mmol) and a solution containing sodium carbonate (0.51 g, 4.77 mmol) dissolved in water (5 ml). The solution was heated for 24 h at 363 K. The white solid that precipitated was collected and recrystallized from acetonitrile–water (1:1) solution to give colorless crystals.

### Refinement

Carbon-bound H-atoms were placed in calculated positions [C–H 0.98 to 1.00 Å, *U*_iso_(H) 1.2 to 1.5*U*_eq_(C)] and were included in the refinement in the riding model approximation.

The amino and hydroxy H-atoms were located in a difference Fourier map, and were refined with distance restraints of N–H 0.88±0.01, O–H 0.84±0.01 Å; their temperature factors were refined.

The (6 0 3) reflection was omitted.

### Crystal data

**Table:**

C_20_H_40_N_4_·2C_11_H_10_O	*Z* = 1
*M**_r_* = 652.94	*F*(000) = 356
Triclinic, *P*1	*D*_x_ = 1.228 Mg m^−^^3^
Hall symbol: -P 1	Mo *K*α radiation, λ = 0.71073 Å
*a* = 8.9706 (4) Å	Cell parameters from 2968 reflections
*b* = 9.4967 (6) Å	θ = 2.8–29.3°
*c* = 10.5580 (5) Å	µ = 0.08 mm^−^^1^
α = 92.500 (4)°	*T* = 100 K
β = 97.961 (4)°	Prism, colorless
γ = 96.666 (4)°	0.30 × 0.25 × 0.20 mm
*V* = 883.13 (8) Å^3^	

### Data collection

**Table:**

Agilent SuperNova Dual diffractometer with an Atlas detector	3915 independent reflections
Radiation source: SuperNova (Mo) X-ray Source	2958 reflections with *I* > 2σ(*I*)
Mirror	*R*_int_ = 0.027
Detector resolution: 10.4041 pixels mm^-1^	θ_max_ = 27.5°, θ_min_ = 2.8°
ω scan	*h* = −11→11
Absorption correction: multi-scan (*CrysAlis PRO*; Agilent, 2010)	*k* = −12→9
*T*_min_ = 0.978, *T*_max_ = 0.985	*l* = −12→13
6926 measured reflections	

### Refinement

**Table:**

Refinement on *F*^2^	Primary atom site location: structure-invariant direct methods
Least-squares matrix: full	Secondary atom site location: difference Fourier map
*R*[*F*^2^ > 2σ(*F*^2^)] = 0.049	Hydrogen site location: inferred from neighbouring sites
*wR*(*F*^2^) = 0.126	H atoms treated by a mixture of independent and constrained refinement
*S* = 1.00	*w* = 1/[σ^2^(*F*_o_^2^) + (0.0516*P*)^2^ + 0.3587*P*] where *P* = (*F*_o_^2^ + 2*F*_c_^2^)/3
3915 reflections	(Δ/σ)_max_ = 0.001
229 parameters	Δρ_max_ = 0.29 e Å^−^^3^
3 restraints	Δρ_min_ = −0.28 e Å^−^^3^

### Fractional atomic coordinates and isotropic or equivalent isotropic displacement parameters (Å^2^)

**Table:**

	*x*	*y*	*z*	*U*_iso_*/*U*_eq_	
O1	0.39115 (13)	0.38946 (12)	0.62565 (11)	0.0246 (3)	
N1	0.10202 (14)	0.30725 (14)	0.49130 (12)	0.0167 (3)	
N2	0.02690 (14)	0.51309 (13)	0.31361 (12)	0.0169 (3)	
C1	0.04567 (17)	0.24648 (16)	0.70859 (14)	0.0187 (3)	
H1A	0.1503	0.2922	0.7383	0.022*	
H1B	0.0293	0.1613	0.7582	0.022*	
C2	0.03614 (18)	0.19676 (16)	0.56804 (14)	0.0195 (3)	
H2A	0.0903	0.1121	0.5622	0.023*	
H2B	−0.0715	0.1684	0.5318	0.023*	
C3	0.07600 (16)	0.26812 (16)	0.35267 (14)	0.0165 (3)	
H3	−0.0335	0.2310	0.3268	0.020*	
C4	0.17260 (18)	0.15236 (17)	0.32043 (15)	0.0218 (3)	
H4A	0.2808	0.1862	0.3509	0.026*	
H4B	0.1442	0.0668	0.3663	0.026*	
C5	0.1525 (2)	0.11239 (18)	0.17690 (16)	0.0274 (4)	
H5A	0.2219	0.0422	0.1601	0.033*	
H5B	0.0473	0.0678	0.1481	0.033*	
C6	0.18552 (19)	0.24225 (18)	0.10128 (15)	0.0238 (4)	
H6A	0.1632	0.2150	0.0084	0.029*	
H6B	0.2943	0.2797	0.1216	0.029*	
C7	0.08938 (17)	0.35713 (17)	0.13392 (14)	0.0191 (3)	
H7A	−0.0191	0.3223	0.1059	0.023*	
H7B	0.1155	0.4420	0.0865	0.023*	
C8	0.11388 (16)	0.39903 (16)	0.27810 (14)	0.0161 (3)	
H8	0.2240	0.4336	0.3040	0.019*	
C9	0.06375 (17)	0.65060 (16)	0.25732 (14)	0.0178 (3)	
H9	0.0493	0.6339	0.1620	0.021*	
C10	0.22827 (17)	0.71117 (17)	0.30174 (16)	0.0237 (4)	
H10A	0.2950	0.6423	0.2792	0.036*	
H10B	0.2441	0.7308	0.3948	0.036*	
H10C	0.2516	0.7994	0.2598	0.036*	
C11	0.40299 (19)	0.53897 (17)	0.62006 (16)	0.0249 (4)	
H11A	0.3248	0.5626	0.5512	0.030*	
H11B	0.5035	0.5735	0.5968	0.030*	
C12	0.38426 (16)	0.61640 (17)	0.74368 (15)	0.0201 (3)	
C13	0.36862 (17)	0.54546 (18)	0.85219 (15)	0.0220 (3)	
H13	0.3698	0.4455	0.8498	0.026*	
C14	0.35081 (18)	0.61807 (19)	0.96724 (16)	0.0256 (4)	
H14	0.3424	0.5668	1.0417	0.031*	
C15	0.34556 (18)	0.76033 (19)	0.97311 (16)	0.0257 (4)	
H15	0.3314	0.8074	1.0510	0.031*	
C16	0.36112 (17)	0.83948 (17)	0.86337 (15)	0.0217 (3)	
C17	0.35874 (19)	0.98840 (19)	0.86739 (17)	0.0276 (4)	
H17	0.3427	1.0366	0.9442	0.033*	
C18	0.37914 (18)	1.06392 (19)	0.76259 (17)	0.0291 (4)	
H18	0.3779	1.1639	0.7669	0.035*	
C19	0.40194 (18)	0.99358 (18)	0.64866 (16)	0.0264 (4)	
H19	0.4160	1.0463	0.5759	0.032*	
C20	0.40408 (17)	0.84951 (18)	0.64119 (15)	0.0230 (4)	
H20	0.4200	0.8038	0.5632	0.028*	
C21	0.38299 (16)	0.76716 (17)	0.74774 (15)	0.0195 (3)	
H1O	0.3056 (15)	0.356 (2)	0.5834 (18)	0.047 (6)*	
H1N	0.0599 (18)	0.3847 (13)	0.5061 (17)	0.027 (5)*	
H2N	−0.0694 (12)	0.4833 (19)	0.2849 (16)	0.028 (5)*	

### Atomic displacement parameters (Å^2^)

**Table:**

	*U*^11^	*U*^22^	*U*^33^	*U*^12^	*U*^13^	*U*^23^
O1	0.0249 (6)	0.0183 (6)	0.0294 (7)	0.0014 (5)	0.0008 (5)	0.0018 (5)
N1	0.0221 (6)	0.0120 (6)	0.0163 (6)	0.0029 (5)	0.0030 (5)	0.0024 (5)
N2	0.0185 (6)	0.0132 (6)	0.0196 (7)	0.0028 (5)	0.0039 (5)	0.0028 (5)
C1	0.0247 (8)	0.0156 (8)	0.0165 (8)	0.0044 (6)	0.0027 (6)	0.0052 (6)
C2	0.0259 (8)	0.0125 (7)	0.0207 (8)	0.0022 (6)	0.0053 (6)	0.0023 (6)
C3	0.0194 (7)	0.0143 (7)	0.0158 (7)	0.0014 (6)	0.0030 (5)	0.0009 (6)
C4	0.0312 (8)	0.0166 (8)	0.0200 (8)	0.0076 (7)	0.0069 (6)	0.0041 (6)
C5	0.0440 (10)	0.0179 (8)	0.0232 (9)	0.0087 (7)	0.0108 (7)	0.0011 (7)
C6	0.0338 (9)	0.0210 (8)	0.0192 (8)	0.0080 (7)	0.0085 (7)	0.0024 (6)
C7	0.0226 (8)	0.0180 (8)	0.0172 (8)	0.0031 (6)	0.0040 (6)	0.0030 (6)
C8	0.0176 (7)	0.0134 (7)	0.0177 (8)	0.0031 (6)	0.0032 (5)	0.0016 (6)
C9	0.0247 (8)	0.0145 (7)	0.0151 (7)	0.0035 (6)	0.0038 (6)	0.0039 (6)
C10	0.0258 (8)	0.0174 (8)	0.0288 (9)	0.0025 (6)	0.0063 (7)	0.0047 (7)
C11	0.0306 (9)	0.0184 (8)	0.0245 (9)	−0.0028 (7)	0.0042 (7)	0.0013 (7)
C12	0.0142 (7)	0.0213 (8)	0.0233 (8)	−0.0010 (6)	0.0000 (6)	0.0016 (6)
C13	0.0194 (8)	0.0211 (8)	0.0242 (8)	−0.0001 (6)	0.0001 (6)	0.0047 (6)
C14	0.0229 (8)	0.0310 (10)	0.0224 (9)	−0.0009 (7)	0.0026 (6)	0.0079 (7)
C15	0.0246 (8)	0.0323 (10)	0.0211 (9)	0.0039 (7)	0.0058 (6)	0.0016 (7)
C16	0.0168 (7)	0.0243 (9)	0.0248 (9)	0.0058 (6)	0.0025 (6)	0.0020 (7)
C17	0.0276 (9)	0.0258 (9)	0.0310 (9)	0.0097 (7)	0.0051 (7)	−0.0012 (7)
C18	0.0266 (9)	0.0209 (9)	0.0400 (11)	0.0090 (7)	0.0002 (7)	0.0032 (8)
C19	0.0246 (8)	0.0253 (9)	0.0287 (9)	0.0034 (7)	−0.0004 (7)	0.0087 (7)
C20	0.0211 (8)	0.0241 (9)	0.0227 (8)	0.0009 (7)	0.0006 (6)	0.0018 (7)
C21	0.0142 (7)	0.0208 (8)	0.0227 (8)	0.0016 (6)	−0.0004 (6)	0.0027 (6)

### Geometric parameters (Å, °)

**Table:**

O1—C11	1.4159 (19)	C8—H8	1.0000
O1—H1O	0.852 (9)	C9—C10	1.522 (2)
N1—C2	1.4730 (19)	C9—C1^i^	1.529 (2)
N1—C3	1.4739 (19)	C9—H9	1.0000
N1—H1N	0.883 (9)	C10—H10A	0.9800
N2—C8	1.4712 (19)	C10—H10B	0.9800
N2—C9	1.4798 (19)	C10—H10C	0.9800
N2—H2N	0.884 (9)	C11—C12	1.509 (2)
C1—C2	1.525 (2)	C11—H11A	0.9900
C1—C9^i^	1.529 (2)	C11—H11B	0.9900
C1—H1A	0.9900	C12—C13	1.370 (2)
C1—H1B	0.9900	C12—C21	1.432 (2)
C2—H2A	0.9900	C13—C14	1.408 (2)
C2—H2B	0.9900	C13—H13	0.9500
C3—C8	1.531 (2)	C14—C15	1.356 (2)
C3—C4	1.531 (2)	C14—H14	0.9500
C3—H3	1.0000	C15—C16	1.422 (2)
C4—C5	1.527 (2)	C15—H15	0.9500
C4—H4A	0.9900	C16—C17	1.416 (2)
C4—H4B	0.9900	C16—C21	1.423 (2)
C5—C6	1.521 (2)	C17—C18	1.366 (3)
C5—H5A	0.9900	C17—H17	0.9500
C5—H5B	0.9900	C18—C19	1.402 (2)
C6—C7	1.523 (2)	C18—H18	0.9500
C6—H6A	0.9900	C19—C20	1.369 (2)
C6—H6B	0.9900	C19—H19	0.9500
C7—C8	1.536 (2)	C20—C21	1.419 (2)
C7—H7A	0.9900	C20—H20	0.9500
C7—H7B	0.9900		
			
C11—O1—H1O	107.1 (15)	N2—C8—H8	107.7
C2—N1—C3	113.32 (11)	C3—C8—H8	107.7
C2—N1—H1N	107.1 (12)	C7—C8—H8	107.7
C3—N1—H1N	109.4 (12)	N2—C9—C10	110.88 (13)
C8—N2—C9	115.46 (11)	N2—C9—C1^i^	109.54 (12)
C8—N2—H2N	106.5 (12)	C10—C9—C1^i^	111.64 (13)
C9—N2—H2N	106.9 (12)	N2—C9—H9	108.2
C2—C1—C9^i^	117.65 (13)	C10—C9—H9	108.2
C2—C1—H1A	107.9	C1^i^—C9—H9	108.2
C9^i^—C1—H1A	107.9	C9—C10—H10A	109.5
C2—C1—H1B	107.9	C9—C10—H10B	109.5
C9^i^—C1—H1B	107.9	H10A—C10—H10B	109.5
H1A—C1—H1B	107.2	C9—C10—H10C	109.5
N1—C2—C1	112.57 (12)	H10A—C10—H10C	109.5
N1—C2—H2A	109.1	H10B—C10—H10C	109.5
C1—C2—H2A	109.1	O1—C11—C12	113.97 (13)
N1—C2—H2B	109.1	O1—C11—H11A	108.8
C1—C2—H2B	109.1	C12—C11—H11A	108.8
H2A—C2—H2B	107.8	O1—C11—H11B	108.8
N1—C3—C8	109.94 (11)	C12—C11—H11B	108.8
N1—C3—C4	110.78 (13)	H11A—C11—H11B	107.7
C8—C3—C4	109.82 (12)	C13—C12—C21	119.27 (15)
N1—C3—H3	108.8	C13—C12—C11	121.36 (15)
C8—C3—H3	108.8	C21—C12—C11	119.36 (14)
C4—C3—H3	108.8	C12—C13—C14	121.18 (16)
C5—C4—C3	112.30 (14)	C12—C13—H13	119.4
C5—C4—H4A	109.1	C14—C13—H13	119.4
C3—C4—H4A	109.1	C15—C14—C13	120.84 (16)
C5—C4—H4B	109.1	C15—C14—H14	119.6
C3—C4—H4B	109.1	C13—C14—H14	119.6
H4A—C4—H4B	107.9	C14—C15—C16	120.42 (15)
C6—C5—C4	111.15 (13)	C14—C15—H15	119.8
C6—C5—H5A	109.4	C16—C15—H15	119.8
C4—C5—H5A	109.4	C17—C16—C15	121.61 (15)
C6—C5—H5B	109.4	C17—C16—C21	119.41 (15)
C4—C5—H5B	109.4	C15—C16—C21	118.96 (15)
H5A—C5—H5B	108.0	C18—C17—C16	121.05 (16)
C5—C6—C7	110.49 (13)	C18—C17—H17	119.5
C5—C6—H6A	109.6	C16—C17—H17	119.5
C7—C6—H6A	109.6	C17—C18—C19	119.87 (16)
C5—C6—H6B	109.6	C17—C18—H18	120.1
C7—C6—H6B	109.6	C19—C18—H18	120.1
H6A—C6—H6B	108.1	C20—C19—C18	120.63 (16)
C6—C7—C8	111.94 (13)	C20—C19—H19	119.7
C6—C7—H7A	109.2	C18—C19—H19	119.7
C8—C7—H7A	109.2	C19—C20—C21	121.27 (15)
C6—C7—H7B	109.2	C19—C20—H20	119.4
C8—C7—H7B	109.2	C21—C20—H20	119.4
H7A—C7—H7B	107.9	C20—C21—C16	117.77 (15)
N2—C8—C3	110.00 (11)	C20—C21—C12	122.93 (14)
N2—C8—C7	113.85 (13)	C16—C21—C12	119.29 (15)
C3—C8—C7	109.72 (12)		
			
C3—N1—C2—C1	−172.40 (12)	C21—C12—C13—C14	0.2 (2)
C9^i^—C1—C2—N1	74.70 (17)	C11—C12—C13—C14	−179.85 (14)
C2—N1—C3—C8	166.81 (12)	C12—C13—C14—C15	1.3 (2)
C2—N1—C3—C4	−71.62 (16)	C13—C14—C15—C16	−1.3 (2)
N1—C3—C4—C5	−178.15 (12)	C14—C15—C16—C17	−178.88 (15)
C8—C3—C4—C5	−56.50 (16)	C14—C15—C16—C21	−0.3 (2)
C3—C4—C5—C6	55.34 (18)	C15—C16—C17—C18	177.67 (15)
C4—C5—C6—C7	−54.34 (19)	C21—C16—C17—C18	−0.9 (2)
C5—C6—C7—C8	56.62 (17)	C16—C17—C18—C19	0.4 (2)
C9—N2—C8—C3	174.49 (11)	C17—C18—C19—C20	0.0 (2)
C9—N2—C8—C7	−61.91 (16)	C18—C19—C20—C21	0.2 (2)
N1—C3—C8—N2	−55.05 (15)	C19—C20—C21—C16	−0.7 (2)
C4—C3—C8—N2	−177.19 (12)	C19—C20—C21—C12	179.92 (14)
N1—C3—C8—C7	178.97 (11)	C17—C16—C21—C20	1.0 (2)
C4—C3—C8—C7	56.83 (16)	C15—C16—C21—C20	−177.60 (14)
C6—C7—C8—N2	178.14 (12)	C17—C16—C21—C12	−179.56 (14)
C6—C7—C8—C3	−58.10 (16)	C15—C16—C21—C12	1.8 (2)
C8—N2—C9—C10	−62.00 (16)	C13—C12—C21—C20	177.61 (14)
C8—N2—C9—C1^i^	174.34 (12)	C11—C12—C21—C20	−2.3 (2)
O1—C11—C12—C13	4.5 (2)	C13—C12—C21—C16	−1.8 (2)
O1—C11—C12—C21	−175.56 (13)	C11—C12—C21—C16	178.28 (13)

Symmetry codes: (i) −*x*, −*y*+1, −*z*+1.

### Hydrogen-bond geometry (Å, °)

**Table:**

*D*—H···*A*	*D*—H	H···*A*	*D*···*A*	*D*—H···*A*
O1—H1o···N1	0.85 (1)	1.94 (1)	2.786 (2)	171 (2)
